# Theranostics in breast cancer

**DOI:** 10.3389/fnume.2023.1236565

**Published:** 2023-08-04

**Authors:** M. Vorster, B. P. Hadebe, M. M. Sathekge

**Affiliations:** ^1^ Department of Nuclear Medicine, University of KwaZulu-Natal, Durban, South Africa; ^2^ Department of Nuclear Medicine, University of Pretoria, Pretoria, South Africa

**Keywords:** theranostics, breast cancer, RLT/radioligand therapy, targeted radionuclide therapy, FAPI, CXCR4

## Abstract

**Introduction:**

Breast cancer is a complex disease and constitutes the leading cause of cancer in women globally. Conventional treatment modalities include surgery, chemotherapy, radiation therapy, and hormonal therapy; all of these have their limitations and often result in significant side effects or toxicity. Targeted radionuclide therapy based on a theranostic approach has been successfully applied in several malignancies, such as prostate cancer, thyroid cancer, and neuro-endocrine tumours. Several studies have also highlighted the potential of theranostic applications in breast cancer.

**Aim:**

This review aims to provide an overview of the most promising current and future theranostic approaches in breast cancer.

**Discussion:**

The discussion includes pre-clinical as well as clinical data on some of the most successful targets used to date. Examples of potential theranostic approaches include those targeting the Human epidermal growth factor receptor 2 (HER2) expression, angiogenesis, aspects of the tumour microenvironment, Gastrin-releasing peptide receptor (GRPR), Prostate-specific membrane antigen (PSMA) and Chemokine receptor 4 (CXCR-4) expression. Several challenges to widespread clinical implementation remain, which include regulatory approval, access to the various radiopharmaceuticals and imaging technology, cost-effectiveness, and the absence of robust clinical data.

**Conclusion:**

Theranostic approaches have the potential to greatly improve diagnosis, treatment, and outcomes for patients with breast cancer. More research is needed to fully explore the potential of such approaches and to identify the best potential targets, considering feasibility, costs, efficacy, side effects and outcomes.

## 1. Introduction

The importance and impact of breast cancer is well known, as it is the most common malignancy to affect women globally (1). This necessitates further exploration of novel, targeted approaches, especially where conventional therapies such as surgery, radiation- and chemotherapy fails. Targeted radionuclide therapies are emerging as a promising theranostic strategy that follows identification of sufficient target expression via imaging as an essential first step (2). The aim of this review is to provide an overview of the current and potential targets for translation into theranostic approaches.

**Figure 1:**
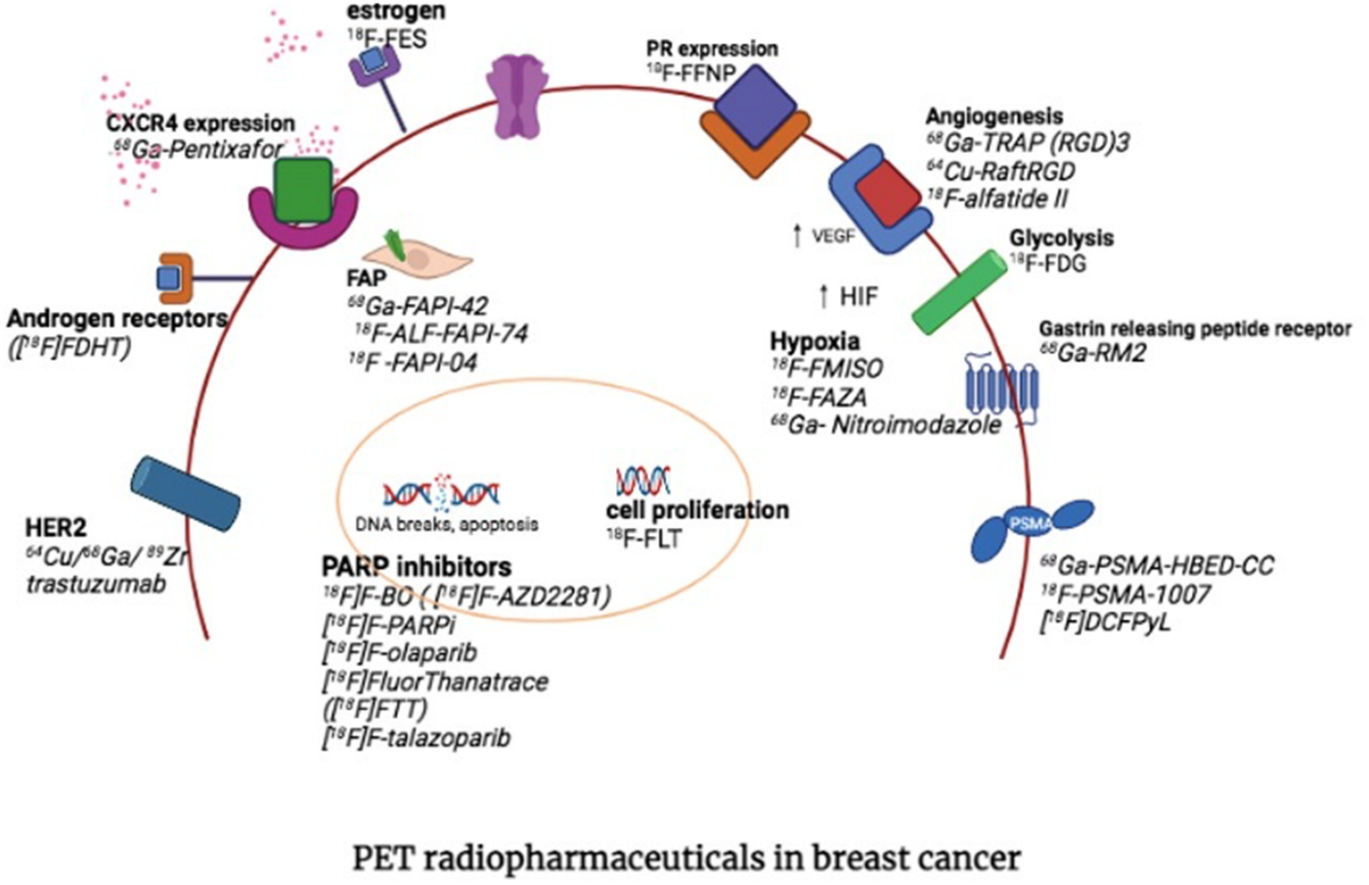
PET targets for theranostic approaches in breast cancer (with permission from Hadebe et al) (7).

Obtaining a clear map of the immunohistopathology and receptor expression, is crucial to selecting and individualising available therapies appropriately. Knowledge of the status of biomarker expression of Estrogen receptors, Progesterone receptors, HER-2 and Ki-67 is integral for accurate subtype classification, direct management, and the best and most appropriate line of therapy (3). The heterogeneity of breast cancer (both intra-and inter-tumoural) however, is well known and unfortunately the treatment plan tends to be largely based on the primary tumour characteristics (4, 5). Considering the impracticality and unacceptability of obtaining multiple biopsies from various disease involvement sites, the advantage of obtaining a whole-body non-invasive investigation with PET/CT first to guide therapy is apparent. Once the therapeutic target is clear, the appropriate isotope can be selected to deliver targeted ionising radiation to cancer cells, whilst sparing healthy cells in what is known as a two-step theranostic approach (6, 7). Figure 1 highlights some of the most important targets in theranostic approaches, which includes Estrogen- and Progesterone receptors, HER-2, GRPR, FAP, PSMA, SSTR, CXCR-4, hypoxia, DNA targets and integrins.

The aim of this review is to summarize some of the most interesting and important pre-clinical and clinical approaches with regards to theranostics in breast cancer and to briefly discuss some of the practical and clinical considerations.

## 2. Human epidermal growth factor receptor 2 (HER2)

### 2.1. Background and rationale

Expression of this receptor occurs in around 20% of patients with breast cancer; it is currently determined by means of biopsy and immunohistochemistry and is considered a negative prognostic factor (8). It is located on the outer cell surface where it is involved in the regulation of cell growth and differentiation. Tumours with HER2 expression tend to be more aggressive and are associated with a relatively high mortality (8, 9).

Trastuzomab (Herceptin™) is a recombinant G1 immunoglobulin monoclonal antibody that targets and blocks the extracellular domain of the HER2 receptor. It is an FDA-approved humanized monoclonal antibody that targets HER2/neu cancer cells, and which causes an inhibitory effect on cell growth (10, 11). It follows then that trastuzomab would make for an attractive pharmaceutical to label with both imaging and therapeutic isotopes.

Non-representative tissue biopsies may unfortunately result in false negatives on immunohistochemistry in up to 20% of cases (12). Considering the potential impact this has on patient management, there is a strong case for PET/CT imaging in determination of HER2 expression.

### 2.2. Pre-clinical evidence

In the pre-clinical setting, studies have made use of the following radiopharmaceuticals, namely ^64^Cu-trastuzomab, ^211^At-trastuzomab, ^111^In-DOTA-trastuzomab and ^177^Lu/^90^Y-trastuzomab, amongst others. These studies (performed on mouse models) have shown mostly promising results with a significant increase in median survival demonstrated (13, 14).

### 2.3. Clinical evidence

A group from Chandigarh, India demonstrated the feasibility of in-house labelling of trastuzomab with Lu-177 via the DOTA chelator. They reported 12 h of stability, and image confirmation of the targeted primary and metastatic lesions was obtained at 5 days post-injection. More recently, the same group shared their findings in 6 patients with HER2 positive metastatic breast cancer compared to four patients with HER2 negative breast cancer (15).

SPECT/CT imaging confirmed uptake in the primary and metastatic lesions in contrast to the HER2 neg group that did not demonstrate any abnormal tracer accumulation. ^177^Lu-trastuzumab administration was well tolerated in nine patients and one patient experienced mild fever. Fever is a known side effect that is associated with trastuzumab administration, and the patient was managed with simple antipyretics. No unexpected toxicities were observed with ^177^Lu-trastuzumab administration and biochemistry, and cardiac function was not affected. Intense liver accumulation is a limiting factor as expected, and further studies are needed. Feasibility of this form of targeted therapy in metastatic HER2 breast cancer was demonstrated (15).

One of the first studies to be published on the use of trastuzomab imaging in patients with breast cancer was by Dijkers and colleagues and involved the use of ^89^Zr-trastuzomab in 14 patients. Normal biodistribution of Zr-89-trastuzumab was reported in the liver, kidneys, spleen, and brain apart from localization in metastatic lesions (16).

Other investigators included Gebhart et al. and Bhusari et al. (15, 17). The latter proceeded to substitute ^89^Zr with Lu-177 in a group of 10 patients with breast cancer to demonstrate feasibility and the possibility of SPECT/CT imaging for target confirmation and dosimetry. They established an optimal imaging time point of 4–5 days post-injection (allowing for sufficient background clearance) and confirmed feasibility of this imaging and therapeutic approach. HER2 positive breast lesions demonstrated sufficient uptake with 10 mCi of ^177^Lu-trastuzumab whilst no uptake was noted on HER2 negative lesions. The authors highlighted the possibility of a therapeutic approach with ^177^Lu-trastuzumab in patients with HER2 positive lesions that are refractory to standard forms of therapy (15).

### 2.4. Treatment considerations

As a complete antibody, trastuzomab is a large protein with a high molecular weight, a long half-life, relatively poor tumour infiltration and inadequate renal filtration. This may lead to limited internalisation and tumour effects, off-target binding with haemato- and hepatotoxicity and inhomogeneous tumour distribution. Most of these problems could, however, be eliminated by making use of an antibody fragment with resultant increased target to background ratio, rapid elimination, and reduced toxicity. The issue of tumour resistance to trastuzomab remains however, and the intense liver accumulation remains problematic (18, 19).

Trastuzomab-directed radionuclide therapy appears feasible and allows for labelling to multiple radioisotopes with promising pre-clinical- and clinical results. The inability to visualise liver lesions due to intense physiological uptake is a limitation.

## 3. Fibroblast activation protein inhibitors (FAPI)

### 3.1. Background and rationale

Cancer-associated fibroblasts (CAF) fulfil an integral role within the tumour micro-environment, where they support and facilitate malignant growth and metastatic spread. These fibroblasts express a protein, FAP-alpha, which provides an attractive target for both imaging and therapeutic translation as it is expressed in the stromal tissues of many malignancies, whilst remaining largely absent from normal tissues. Considering that the stroma can account for up to 90% of the total tumor mass, this may increase tumor cell accessibility for pharmacologic, immunologic, or cell-based therapies. Simultaneous irradiation of CAFs and surrounding tumor cells may also be achieved through the crossfire effect. Additionally, since FAP-expressing CAFs are known to be immunosuppressive, a combination with immunotherapy may produce a synergistic therapeutic effect (20, 21).

Fibroblast Activation Protein is a type II integral membrane glycoprotein from the serine protease family that plays a role in fibrogenesis and in the remodelling of the extracellular matrix. FAP-inhibitors (derived from quinoline peptidomimetics) are used to target these receptors and several forms of FAPI are available, such as FAPI-04, FAPI-02, FAPI-46, FAPI-2286. Major advantages offered by this target include its superior target to background ratios, the lack of any required patient preparation and its potential for therapeutic translation (22–24).

Fibroblast Activation Protein as a theranostic target has enjoyed significant attention in recent times with a 2023 review article including 35 publications on targeted radioligand therapy in both the pre-clinical and clinical settings and stating that over 100 patients have been treated with different FAP targeted radionuclide therapies (including ^177^Lu-FAPI-04, ^90^Y-FAPI-46, ^177^Lu-FAP-2286, ^177^Lu-DOTA.SA.FAPI and ^177^Lu-DOTAGA.(SA.FAPi)2. These studies have reported objective responses in difficult to treat end-stage cancer patients with manageable adverse events (25).

### 3.2. Clinical evidence

Ballal and colleagues shared their experience in a 2020 publication on the compassionate use of ^177^Lu-DOTA-FAPi in a 31-year-old female with breast cancer and a metastatic brain lesion. The primary breast tumour was characterised as ER-, PR- and HER 2+, and she had disease progression on conventional therapy. They administered 3.2 GBq of ^177^Lu-DOTA-FAPi with target lesion confirmation on dosimetry images, which resulted in symptomatic relief.

A later study with SA.FAPI reported on a total of ten patients, which included four with breast cancer (26).

Baum and colleagues also reported on the use of 5.8 =/− 2 GBq of ^177^Lu-FAPI-2286 in eleven patients with a variety of malignancies that included four patients with breast cancer. Treatment in this group was well tolerated with acceptable dosimetry and toxicity in addition to resulting in significant pain relief. Post-therapy images revealed significant uptake of ^177^Lu-FAP-2286 and long retention of the radiopharmaceutical in all patients (27). In contrast, FAPI-02 and FAPI-04 demonstrated earlier washout with a corresponding shorter tumour retention time (28).

Lindner et al. used 2.9 GBq of ^90^Y-FAPI-04 to treat a patient with metastatic breast cancer, which resulted in significant pain reduction without significant side effects. The authors postulated that higher treatment doses could potentially be used to increase the tumoricidal effect. Bremsstrahlung images confirmed the intended target lesion delivery up to one day post-injection (28).

Assadi et al. treated 21 patients (including 5 breast cancer patients) with advanced stage malignancies with 1.85–4.44 GBq of ^177^Lu-FAPi-46 per cycle. Feasibility and an acceptable side effect profile was demonstrated in this compassionate use setting (29).

The LUMIERE trial is a phase I/II study to evaluate the safety, dosimetry, pharmacokinetics, and preliminary anti-tumor activity of ^177^Lu-FAP-2286 in patients with advanced or metastatic solid tumours (30).

A dosimetry study by Kuyumcu et al. following administration of ^177^Lu-FAPI-04 to four patients (which included one patient with breast cancer) also highlighted the short tumour retention time of FAPI-04 whilst confirming acceptable dosimetry (31).

These initial experiences highlight the safety and feasibility of using radiopharmaceuticals such as ^90^Y-FAPI-04 and ^177^Lu-DOTA.SA.FAPI in settings where patients are refractory to conventional forms of therapy.

### 3.3. Treatment considerations

Several FAP-based tracers have been reported with most data referring to FAPI-04, FAPI-46, FAP-2286, and DATAGA.(SA.FAPI)2, and promising clinical data reported in patients with breast cancer.

Studies have reported acceptable side effect and toxicity profiles, with the most important reported adverse events related to bone marrow toxicity (anaemia, leukopenia, and reduced number of platelets), similar to the now known side effect profiles of ^177^Lu-PSMA and ^177^Lu- DOTATATE. Based on the inconsistent data from the various small and non-standardised studies, it is not yet possible to conclusively determine which FAP inhibitor has the best efficacy and toxicity profile. The reported absorbed dose to the tumor are comparable to those of ^177^Lu-DOTATATE and ^177^Lu-PSMA-617 and range from 0.62 ± 0.55 Gy/GBq, 2.81 ± 1.25 Gy/Gbq, 3.0 ± 2.7 Gy/GBq, and 6.70 (IQR: 3.40–49) Gy/GBq, for ^177^Lu-FAPI-04, ^90^Y-FAPI-46, ^177^Lu-FAP-2286, and ^177^Lu-DOTAGA.(SA.FAPi)2, respectively (32, 33).

Most malignancies express FAP in the tumour micro-environment, while some cancers express FAP on their cellular membrane (e.g., sarcoma, certain ovarian, and pancreatic cancers). Crucial to the success of FAP-based targeted radionuclide therapy is the issue of tumour retention time, which should be long enough to achieve a tumoricidal effect, without increasing radiation to non-target lesions and organs. To date the most promising results have been reported with ^177^Lu-FAP-2286, ^177^Lu-DOTAGA.(SA. FAPi)2, ^177^Lu-PNT6555, and the albumin-binding tracers (e.g., ^177^Lu-EB-FAP) which demonstrates uptake up to 168-h post injection. Attempts to increase tumour retention time have focused on labelling FAPi to radio-isotopes with shorter half-lives (such as Y-90, ^188^Re, ^153^Sm, ^213^Bi, or ^212^Pb) in an attempt to better match the peptide half-life to that of the isotope. Other strategies to improve on dose delivery to the tumour include development of FAPI-derivatives with better pharmacokinetics and substituting b-emitters with alpha emitters (34).

A publication by Mori summarizes the most important challenges to be addressed with FAPi-based radioligand therapy as follows: (1) increasing tumor retention time to achieve sufficient cumulative activity in the tumor to facilitate biologically effective radiation doses, (2) selection of the most appropriate radionuclide to match the physiological and biological half-life of the tracer, (3) heterogeneity, and (4) minimizing radiation toxicity (35).

## 4. Prostate-specific membrane antigen (PSMA)

### 4.1. Background and rationale

Prostate-specific membrane antigen (PSMA) is a type 2 transmembrane protein that is encoded by the FOLH1 (folate hydrolase 1) gene and is overexpressed not only in prostate cancer cells but also in the neo-vasculature of several solid tumours such as breast cancer. Preclinical data suggest that PSMA may be involved in cancer-related angiogenesis through degradation of the extracellular matrix and by participating in integrin signal transduction. PSMA expression has been closely linked both to enhanced angiogenesis and tumour aggressiveness. Interestingly, estrogen and progesterone-negative breast tumours tend to exhibit a higher PSMA expression than receptor-positive phenotypes. In the setting of triple negative breast cancer, this becomes especially useful as therapeutic receptor targets are absent and expression has been demonstrated to increase in hypoxic conditions (36, 37).

### 4.2. Preclinical evidence

A 2023 *in vitro* study publication by Heesch and colleagues analysed PSMA and its isoform expression in Triple Negative Breast Cancer cells, breast cancer stem cells, and tumor-associated endothelial cells. They found PSMA expression in 91% of the investigated Triple Negative Breast Cancer cell lines and highlighted the possible role of therapeutic strategies in this clinical setting. The researchers demonstrated that TNBC cells strongly induce PSMA in tumor-associated vasculature, which makes it a promising candidate to target tumor angiogenesis. Interestingly, they also found that hypoxia strongly increased PSMA expression in breast cancer cells, which could be approached by administering PSMA therapy in a hypo-fractionated way, with the first fraction targeting PSMA-expressing endothelial cells and, in this way, decreasing the oxygen supply (38).

Morgenroth et al. used a mouse model of triple negative breast cancer to evaluate the potential of prostate-specific membrane antigen (PSMA) as a target for radio-ligand therapy. The researchers used ^177^Lu-PSMA-617 and their results demonstrated that it strongly impaired the vitality and angiogenic potential of human umbilical vein endothelial cells. Target lesion localisation was confirmed and the authors concluded that ^177^Lu-PSMA-617 could potentially be used in a theranostic approach (39).

In tumours other than prostate cancer, PSMA expression happens mainly in the endothelial cells of tumor-associated neo-vasculature, with no endothelial expression under physiological conditions (40).

### 4.3. Clinical evidence

In a 2013 study by Wernicke et al, the researchers set out to evaluate PSMA expression in tumour-associated vasculature in 106 patients with invasive breast cancer ranging from stage I–IV. Representative tumour tissue from each participant was stained for PSMA expression together with CD31 for confirmation of vasculature presence. The level of PSMA expression detected was scored from 0 to 2 in the following way: 0 = no detectable expression up to 2 = presence of PSMA expression in >50% of micro vessels. PSMA expression was present in 74% of primary breast cancer tumours and in all (*n* = 14) of those with brain metastases. Furthermore, its expression was absent in 98% of normal breast tissue and associated vasculature. Their results further demonstrated that patients with the highest PSMA expression (score of 2) demonstrated higher median tumour size, higher Ki-67, and poorer overall survival. Patients in whom Estrogen receptors were negative were more likely to have a PSMA score of 2 (41).

In a study consisting of 19 patients with breast cancer, Sathekge et al. demonstrated significant PSMA uptake in 84% of the 81 malignant and metastatic lesions with ^68^Ga-PSMA-HBED-CC PET/CT. Metastatic lesions demonstrated higher SUV mean values compared to the primary tumours and no specific association with the presence or absence of progesterone receptors could be demonstrated. The authors highlighted the potential role in anti-angiogenic therapeutic strategies (42).

In one of the biggest studies to date, Tolkach and colleagues evaluated PSMA expression in the tumour endothelium (via immunohistochemistry) of 315 breast cancer patients with invasive non-special type carcinoma (NST) and lobular carcinoma. This group demonstrated PSMA expression in 60% of their study population, with higher expression noted in higher grade tumours and those with HER-2 expression and the highest expression in those with triple negative breast cancers (43).

A 2021 review article by Uijen et al, summarizes the use of PSMA-based radioligand therapy in solid tumours other than prostate cancer, which included breast cancer. The authors summarised the PSMA expression (based on SUVmax values) as low to moderate in breast cancer and did not highlight breast cancer as an ideal candidate for treatment with PSMA-based radioligand therapy (40).

In one of the few published cases of PSMA-based targeted radionuclide therapy in breast cancer, Tolkach et al. shares their experience in treating a young woman with an aggressive triple negative breast cancer that was unresponsive to several conventional lines of therapy. Two cycles of Lu-177-PSMA-RLT at a dose of 7.5 GBq was administered based on sufficient PSMA expression on ^68^Ga-PSMA PET/CT. Post-injection SPECT imaging confirmed delivery of the radiopharmaceutical to the targeted lesions and no adverse effects were reported. Treatment was discontinued after the second cycle of therapy based on disease progression (43).

### 4.4. Treatment considerations

Considering the beta particle tissue range (+/−2 mm) of radioligand therapy with ^177^Lu-PSMA, it could potentially still reach nearby tumour cells in well-perfused malignancies, even if PSMA is exclusively over-expressed in the endothelial cells of neo-vasculature, with resultant abscopal effect. Other concerns with ^177^Lu-PSMA therapy that targets PSMA expression only on the neo-vasculature relate to the expected short tumour retention time and reduced efficacy.

## 5. Somatostatin receptors (SSTRs)

Somatostatin receptor expression occurs in breast cancer whenever there is neuro-endocrine differentiation. This demonstrates overexpression of mostly SSTR2A and SSTR5 on immunohistochemistry and is associated with a worse prognosis (44). Kumar et al. used RT-PCR and immunohistochemistry to investigate the SSTR subtype expression in breast cancer and reported the following levels of expression: SSTR1 (84%), SSTR2 (79%), SSTR3 (89%), SSTR4 (68%), and SSTR5 (68%). Estrogen receptor (ER) levels were seen to correlate with SSTR 1, 2, and 4, while a correlation with progesterone receptor (PR) levels was seen only with SSTR2 (45).

A study by Zou et al. on 160 specimens of ductal breast carcinoma reported SSTR expression as follows: SSTR1 (90%), SSTR4 (71.3%), SSTR5 (44.4%), SSTR3 (41.9%), and SSTR2 (34.4%). They found that SSTR expression was inversely correlated to tumor differentiation, and there was no association between receptor expression and hormone receptors (ER/PR). These variations highlight the need for further studies to elaborate on the expression of SSTR and ER/PR hormone receptors (46).

### 5.1. Preclinical studies

Dalm et al. targeted SSTR2 receptors in a mouse model of breast cancer with both an SSTR agonist (^177^Lu-DOTATATE) and an antagonist (^177^Lu-DOTA-JR11). Results demonstrated a five times higher tumour accumulation of ^177^Lu-DOTA-JR11 compared to the agonist target and this uptake translated to better tumour control and survival rates in mice (47).

### 5.2. Clinical studies

Savelli and colleagues evaluated the use of ^90^Y-DOTATOC in a patient with metastatic breast cancer with neuroendocrine differentiation and liver metastases. They administered 2.57 GBq of ^90^Y-DOTATOC over a course of three cycles and used bremsstrahlung to confirm the intended target with SPECT/CT at 4 days post-administration. They reported a significant CT and biochemistry response in the absence of any adverse effects (48).

In a case study published by Liu et al., the authors administered 6 cycles of ^177^Lu-DOTATOC in a patient with metastatic invasive ductal breast carcinoma and primary large-cell neuroendocrine breast cancer. Intense tracer accumulation was confirmed in the target lesions, which led to a partial treatment response in the absence of any significant side effects or toxicity (49).

The current level of evidence is unfortunately mostly limited to pre-clinical studies and isolated clinical case reports.

## 6. Chemokine receptor 4 (CXCR-4)

The CXCR4 chemokine (in association with CXCL12) is actively involved in processes that support growth and metastatic spread, such as gene expression, cell proliferation and migration, tumorigenesis, and angiogenesis. Increased CXCR4 is strongly associated with a shorter overall survival and a shorter progression-free survival. Patients who are eligible for CXCR4-directed radioligand therapy may be treated with β-emitters such as ^177^Lu/^90^Y-PentixaTher, and this approach has been used successfully mainly in the clinical settings of hematological malignancies (50).

CXCR4-directed therapies have the potential to provide therapeutic options to breast cancer patients without sufficient expression of hormonal receptors or those who become treatment resistant. The feasibility of such an approach has been demonstrated in pre-clinical studies with successful inhibition of cancer progression and metastases.

An example of a selective CXCR4 antagonist is balixafortide, which has been shown to enhance the cytotoxic effect of chemotherapy in breast cancer. Pernas et al. demonstrated the safety and tolerability of balixafortide in combination with eribulin in 56 heavily pre-treated patients with relapsed HER2-negative metastatic breast cancer (51). These findings were subsequently confirmed in the phase 3 randomized multicentre FORTRESS trial with objective responses documented in 35% of patients (52).

Injected ^177^Lu-Pentixfor binds to plasma proteins with high stability, leaving less than 5% available to bind to CXCR4 receptors on white blood cells and platelets. Radiopharmaceutical accumulation takes place in CXCR4-expressing malignant and metastatic lesions (with high tumour retention) as well as in the liver, kidneys, and bone marrow. Tracer accumulation in the bone marrow has a half-life of several days, which makes this the critical organ for acute toxicity. Bone marrow doses range from 0.14 to 2.3 (median value, 0.5) Gy/GBq ^177^Lu, which makes it suitable for myeloablative therapy; even in this setting, Lu-177 may be best substituted with the shorter-lived Y-90. Reports on renal dosimetry (as the dose-limiting organ) also vary greatly, ranging from 0.14 to 2.3 (median value, 0.5) Gy/GBq ^177^Lu (53).

Considering the promising results that have been obtained with CXCR4-directed radioligand therapy in hematological malignancies, the same approach could potentially be applied to solid tumor patients with sufficient CXCR4 expression on PET. However, optimal bone marrow support (stem cell transplant) would likely be required.

## 7. Gastrin-releasing peptide receptor (GRPR)

This transmembrane G-protein coupled receptor can be conjugated to bombesin and provides several attractive agonist and antagonist possibilities for both imaging and therapeutic possibilities. Gastrin-releasing peptide receptor (GRPR) is overexpressed in over 75% of breast cancers and can be efficiently targeted with ^177^Lu-Bombesin to deliver therapeutic radiation doses to malignant cells. Bombesin is an analog of the mammalian gastrin-releasing peptide (GRP) and has demonstrated high sensitivity to detect the GRP receptor in breast cancer (54).

The bombesin agonist AMBA, linked to DOTA, as ^177^Lu-AMBA was first reported to demonstrate high therapeutic potential in several prostate cancer models with different levels of GRPR expression. Other therapeutic labelling options include Terbium and Scandium's therapeutic isotopes as well as alpha emitters such as Ac-225 and Bi-213.

Initial studies with radiotracers labelled to GRPR agonists highlighted some unfavourable characteristics related to low metabolic stability, unfavourable abdominal accumulation, and adverse effects, which motivated a change toward GRPR antagonists. These safer analogues demonstrate improvements in metabolic stability and background clearance.^68^Ga-RM2 is an example of such a GRPR antagonist that is one of the more frequently used imaging possibilities (55).

In a 2016 pre-clinical feasibility study, Aranda-Lara and colleagues demonstrated high tumour uptake of ^177^Lu-Bombesin in a mouse-model. More recent preclinical studies in mice models have demonstrated the increased efficacy of the mTOR inhibitor rapamycin when used in combination with ^177^Lu-RM2 (56, 57).

### 7.1. Clinical studies

A first in human study evaluated the use of ^177^Lu-RM2 in the setting of metastatic castration-resistant prostate cancer. Tumour absorbed doses were found to be therapeutically relevant, whereas rapid clearance from normal GRPR-rich organs, such as the pancreas, was confirmed. (The latter is considered to be the dose-limiting organ due to its high radioligand uptake). Despite the encouraging dosimetry results, certain derivatives demonstrate poor metabolic stability, which limits theranostic use (56). Lu-177-AMTG was identified as the therapeutic candidate with the most potential, but further studies are needed. ^68^Ga/^177^Lu-NeoBOMB1 provides another promising theranostic approach which has been evaluated in prostate cancer patients with good detection of primary tumours and liver and bone metastases (58).

Combination therapies with mTOR-inhibitors, PRRT, immunotherapy and hormone therapy in breast cancer may provide another promising route toward higher therapeutic responses.

## 8. Poly-ADP ribose polymerase (PARP)

Poly-ADP ribose Polymerase-1 (PARP1) is a nuclear DNA repair enzyme which functions as an early sensor of single strand DNA breaks. Breast Cancer proteins (BRCA1/2mut) may result in faulty double strand DNA repair mechanisms, leading to PARP1-mediated processes. Rapidly proliferating cancer cells are under higher replicative stress, which leads to genomic instability and overexpression of PARP1. PARP-1 inhibition presents a target for theranostic approaches in breast (and ovarian) cancer (59).

Imaging of PARP1 expression may identify patients who are likely to respond to PARP inhibitor treatment; it may provide prognostic information and it has the potential to provide personalised and targeted treatment options. Examples of FDA-approved PARP inhibitors (PARPis) include olaparib, rucaparib, niraparib and talazoparib (60, 61).

### 8.1. Preclinical studies

Sankaranarayanan et al, investigated a theranostic approach in a triple negative breast cancer xenografted mouse model by radiolabelling the PARPi-derivative Olaparib to the Auger emitters ^123/125^I. SPECT/CT images were acquired at 4 h or 24 h post injection of ^123^I-PARPi-01, followed by a therapeutic approach with ^125^I-PARPi-01 administered in 4 doses of 10 MBq/dose injected 10 days apart. A significant delay in tumour growth and doubling time was demonstrated on weekly CT scans, in the absence of significant radiotoxicity to the thyroid and liver. A significantly higher apoptosis ratio was demonstrated in the ^125^I-PARPi-01-treated tumour tissues (62).

This presents a potential targeted radionuclide therapy approach for patients with triple negative breast cancer, although improvements to the pharmacokinetics are necessary prior to its potential clinical application.

## 9. Integrin imaging with arginine-glysine-aspartic acid (RGD)

Angiogenesis with integrin expression is an essential component of malignant growth and metastatic spread. Integrin *α*vβ3 is a receptor for extra-cellular matrix proteins with the exposed arginine(R)-glycine(G)-aspartic acid(D) tripeptide sequence, and as such it provides an interesting molecular target for both diagnostic and therapeutic approaches in solid tumours (63).

Apart from detecting areas of neo-angiogenesis, RGD uptake values on a baseline PET study can be used to predict how likely a tumour is to respond to antiangiogenic therapy (64, 65). Furthermore, it can be used to monitor the occurrence of adverse events in normal organs.

In diagnostic PET/CT studies, researchers have generally demonstrated lower values of RGD accumulation when compared to FDG, with superior brain lesion detection of RGD but inferior detection of liver involvement. RGD uptake varies significantly depending on hormonal receptor expression in breast cancer. ^18^F-Alfatide II has for example demonstrated higher uptake than ^18^F-FDG in HER2 (–) and ER(+) breast cancer lesions. ^68^Ga-BBN-RGD represents another example of a RGD-based PET/CT tracer (66–68).

Both ^18^F-Alfatide II and ^68^Ga-BBN-RGD offer good diagnostic performance in the detection of breast cancer, which is comparable to ^18^F-FDG but not necessarily superior. RGD-based tracers may however have superior detection in breast cancer that expresses estrogen receptors in the absence of HER2 (67, 68).

In a theranostic approach, ^68^Ga may be replaced with ^177^Lu in order to target lesions with integrin overexpression, as has been successfully demonstrated in pre-clinical studies with ^177^Lu-EB-RGD in the setting of lung cancer. Clinical translation in the setting of breast cancer may potentially enable a highly personalised approach using ^68^Ga-RGD PET/CT to non-invasively image and quantify integrin *α*vβ3 expression and accordingly select the most suitable patients who are likely to benefit from therapy (69).

## 10. Radiopharmaceutical choice

Mapping and identification of the primary tumour characteristics is crucial in directing selection of the most appropriate imaging and therapeutic targets. Therapeutic isotope possibilities include both alpha- and beta emitters, such as I-131, Lu-177, Y-90, Ac-225, Ra-223 and Tb-161 amongst others, and these will likely be selected based on a combination of factors that include cost, availability, logistics, tumour size and distribution patterns.

The beta-emitter Lu-177 is currently the most widely used therapeutic radioisotope after ^131^I following its excellent track record in the settings of prostate cancer and neuroendocrine tumours. Other advantages include the relatively long half-life (to allow transport and other logistics), favourable nuclear decay characteristics, chemistry that allows for versatile labelling options, high radiochemical yield and stability with DOTA-derivatives and the post-treatment imaging possibility for target confirmation and dosimetry.

Alpha emitters provide the advantages of a high linear transfer energy and high tumoricidal- and bystander effects that are independent of oxygen supply and cell cycle timings. Disadvantages include access, high costs and reliable supply in addition to problematic dosimetry assessments. ^225^Actinium have been tested since 2016 with encouraging results in prostate cancers amongst others.

Therapeutic isotopes should be selected based on the range of radiation required in tissue with appropriate matching of the half-life to that of the linked peptide, whilst considering the safety profile in the context of the patient's existing organ function and co-morbidities.

## 11. Conclusions

Despite advances in the treatment of breast cancer, there is still a significant portion of the breast cancer population who are not ideally suited to, or who ultimately fail, currently available therapies and are subsequently left with few or no options. It is this group that may potentially benefit most from targeted radioligand therapies chosen to suit the individual patient's particular tumour characteristics and receptor expression. Notwithstanding increasing reports on the therapeutic use of various radiolabeled targets in breast cancer, evidence remains limited to small, non-standardized observational studies and to isolated case reports. Theranostic approaches have the potential to greatly improve diagnosis, treatment, and outcomes for patients with breast cancer. This review highlights the need for further research to explore the potential of such approaches and to identify the best potential targets, whilst considering access, feasibility, costs, efficacy, side effects and outcomes.
